# A case of sacrococcygeal teratoma associated with antenatally acquired urethrovaginal fistula and hydrocolpos

**DOI:** 10.1186/s40792-023-01772-y

**Published:** 2023-10-31

**Authors:** Yuichi Shibui, Satoshi Obata, Ryuichiro Hirose, Ryo Nakano, Takashi Setoue, Takeshi Miyazaki, Hirofumi Matsuoka, Toshihiko Sato

**Affiliations:** 1 Department of General Thoracic, Breast, and Pediatric Surgery, Fukuoka University, 7-45-1, Nanakuma Jonan-Ku, Fukuoka, Fukuoka 814-0180 Japan; 2 Department of Pediatric Surgery, Faculty of Medicine, University of Tsukuba, 1-1-1, Tennodai, Tsukuba, Ibaraki 305-8575 Japan; 3 Division of Neonatology, Center for Maternal, Fetal and Neonatal Medicine, Fukuoka University Hospital, 7-45-1, Nanakuma Jonan-Ku, Fukuoka, Fukuoka 814-0180 Japan; 4 Department of Urology, Faculty of Medicine, Fukuoka University, 7-45-1, Nanakuma Jonan-Ku, Fukuoka, Fukuoka 814-0180 Japan

**Keywords:** Sacrococcygeal teratoma, Urethrovaginal fistula, Hydrocolpos, Urological complications, Urogenital sinus

## Abstract

**Background:**

Sacrococcygeal teratomas (SCTs) are known to cause urological complications, but urethrovaginal (UV) fistula as a complication of SCT is rare. We herein report a case of SCT with UV fistula and hydrocolpos.

**Case presentation:**

A 1-day-old female neonate presented to our department with prominent swelling in the sacrococcygeal region. She was born at 37 gestational weeks via spontaneous vaginal delivery from a 39-year-old woman. The weight of the baby was 2965 g, and her Apgar scores were 4/10 (at 1 and 5 min). An MRI examination confirmed an 11 × 11 cm Altman classification typeII SCT associated with hydrocolpos, a dilated urinary bladder, and bilateral hydronephrosis. When she was 5 days, the SCT was excised totally and a coccygectomy was performed. After the operation, as her urinary output appeared unstable, a cystoscopic examination was performed on the third postoperative day. This revealed that the UV fistula was located approximately 1 cm from the urethral opening. In addition, the proximal urethra was unobstructed and connected to the bladder. The cystoscope allowed for the passage of a urinary catheter through the urethra. After 1 month of catheter placement, she was discharged from the hospital at 57 days of age. Follow-up was uneventful, with neither urinary infection nor retention.

**Conclusions:**

SCTs are associated with not only trouble with rectal function and lower extremity movement but also urinary complications. The pathogenesis of this UV fistula is thought to be the rapid growth of the SCT that developed in the fetal period, resulting in obstruction of the urethra by the tumor and the pubic bone, which in turn caused urinary retention and the formation of a fistula as an escape route for the pressure. Because SCTs can cause a variety of complications depending on the course of the disease, careful examination and follow-up are necessary.

## Introduction

Sacrococcygeal teratomas (SCTs) are among the most common fetal and neonatal tumors, with an incidence of about 1 in every 14,000–40,000 live births [1, 2]. SCTs are known to cause urological complications, such as urinary retention, hydronephrosis, and neurogenic bladder [3]. Urethrovaginal fistula (UV) is also a complication of SCT, but a rare one. We herein report a case of a newborn with an antenatally acquired UV fistula and hydrocolpos associated with type-II SCT.

## Case

A 1-day-old female neonate presented to our department with prominent swelling in the sacrococcygeal region. As her parents during the pregnancy had elected not to learn the sex of the baby in advance, ultrasound examination had not included the fetal public region. However, fetal hydrops and/or abnormal amniotic fluid volume had not been found throughout the prenatal checkup.

The baby was born at 37 gestational weeks via spontaneous vaginal delivery from a 39-year-old woman. At the time of delivery, the baby weighed 2965 g and the Apgar scores were 4/10 at 1 min. Neither meconium staining nor nuchal chord was observed. After oxygen administration and bagging, the Apgar scores were improved to 9/10 at 5 min.

**Fig. 1:**
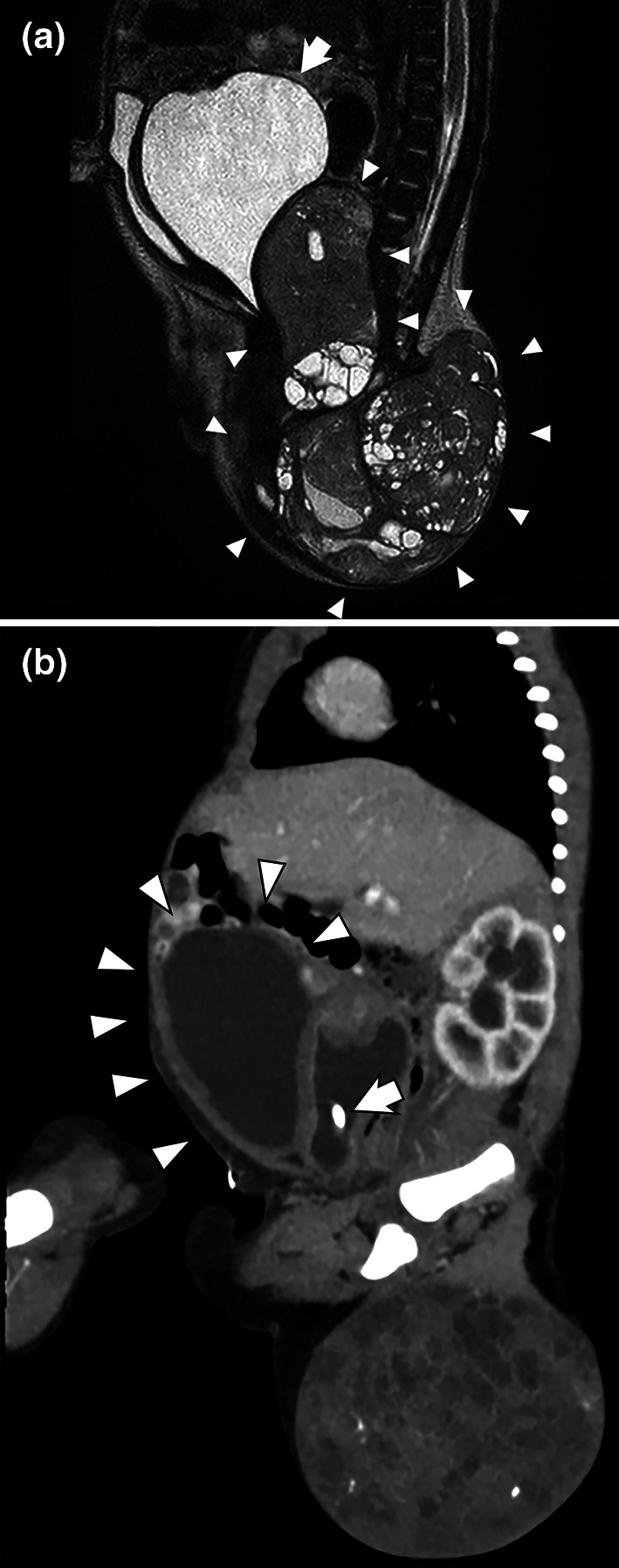
**a** Sagittal T2-weighted magnetic resonance imaging at 1 day of age through the pelvis shows an 11 cm mass indicating Altman’s type II sacrococcygeal teratoma (arrowhead) and hydrocolpos (arrow). **b** Computed tomography imaging at 2 days of age shows a shrunken hydrocolpos, an inversely dilated bladder (arrowhead), and bilateral grade 2 hydronephrosis. An indwelling catheter is inserted into the vaginal cavity (arrow)

Laboratory investigations were conducted. Her hemoglobin was 15.9 g/dl, alpha fetoprotein was 1.83 × 10^5^ ng/ml, and the other results were within normal limits. Ultrasonography over the sacrum revealed a mostly solid tumor. MRI at 1-day-old confirmed an 11 × 11 cm sacrococcygeal tumor associated with hydrocolpos, a slightly dilated urinary bladder (Fig. 1a), and bilateral hydronephrosis. From these findings, she was diagnosed with Altman classification typeII SCT.

After admission, she showed difficulty urinating, so we tried to insert an indwelling urinary catheter (IUC) via her urethral orifice, but the catheter passed into the vaginal cavity rather than into the bladder. Residues caused the repeated obstruction of the indwelled catheter, but urine intermittently flowed out through the outer part of the catheter. Daily urinary output was 359 ml at 2 days and 273 ml at 3 days.

Computed tomography at 2 days showed a shrunken hydrocolpos with an inversely dilated urinary bladder and bilateral grade 2 hydronephrosis (Fig. 1b).

**Fig. 2:**
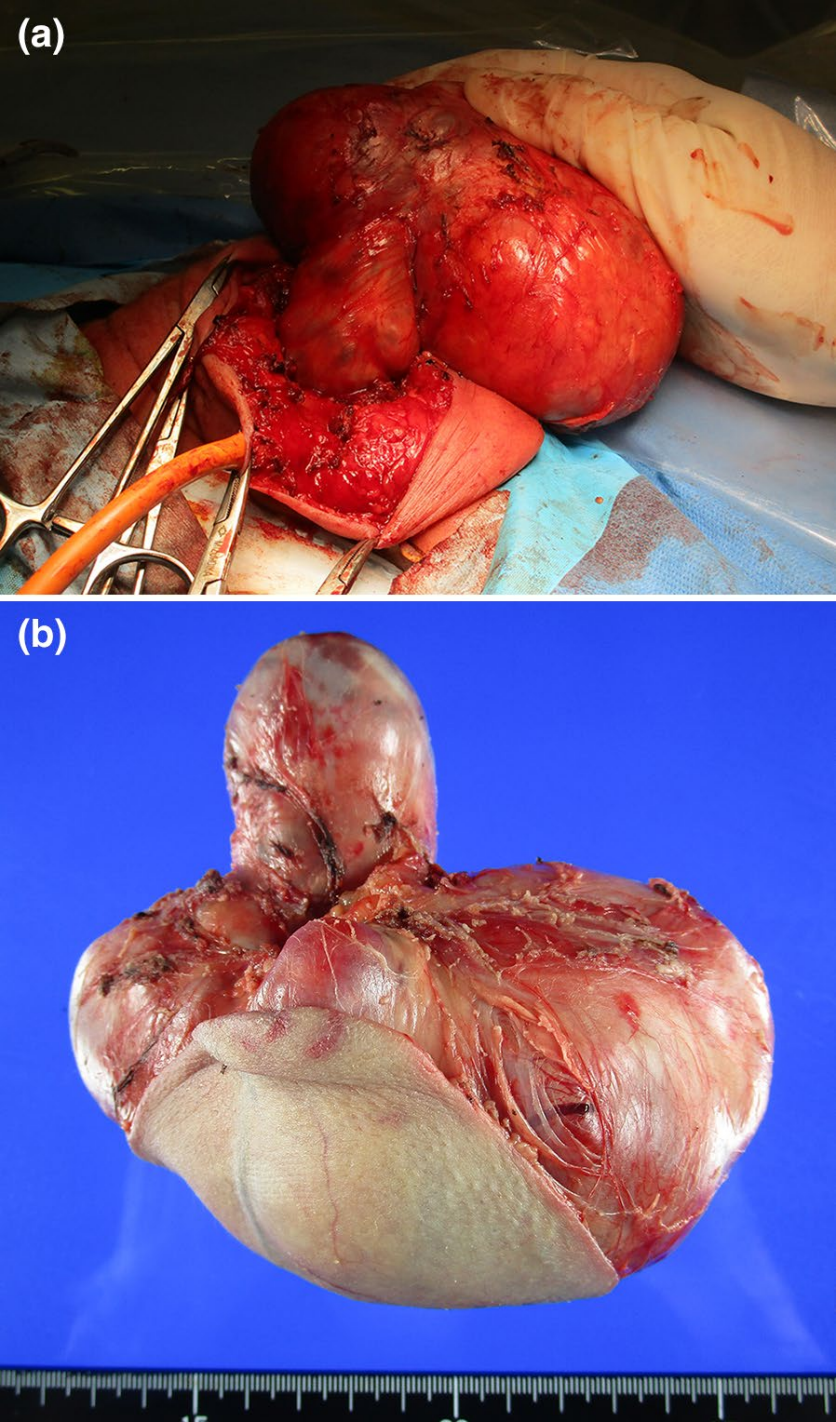
SCT was excised totally along with a coccygectomy through an abdominal and posterior approach

**Fig. 3:**
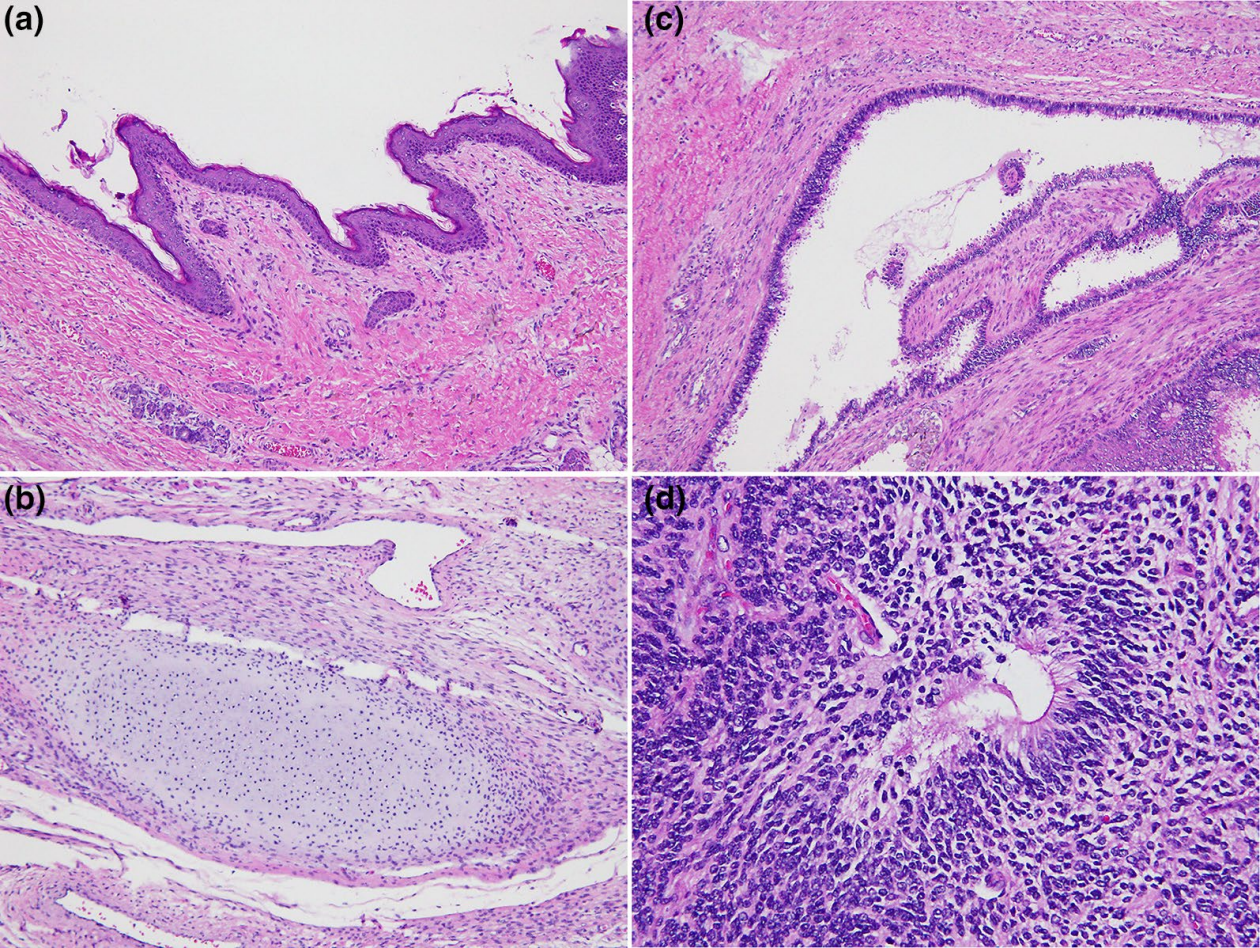
Pathological findings revealed various mature tissues, such as skin, its appendages (H&E, × 200, **a**), choroid plexus, bone, cartilage (H&E, × 200, **b**), and ciliated epithelium (H&E, × 200, **c**) with foci of immature neuroepithelium (H&E, × 400, **d**)

At 5 days of age, the SCT was excised totally along with a coccygectomy. First, we ligated a median sacral artery, which was a feeding vessel of the tumor and exfoliated the tumor as much as possible through an abdominal approach. We then performed en bloc tumor excision, including the coccyx, using a posterior approach (Fig. 2a, b). Throughout the operation, there were no injuries to the urethra or rectum. Pathological findings revealed mostly mature tissue elements originating from all 3 germ layers with foci of immature neuroepithelium, consistent with grade 2 immature teratoma (Fig. 3a–d).

After the operation, urine flowed intermittently to the diaper, but urinary output appeared unstable. Therefore, a cystoscopic examination was performed on the third postoperative day.

**Fig. 4:**
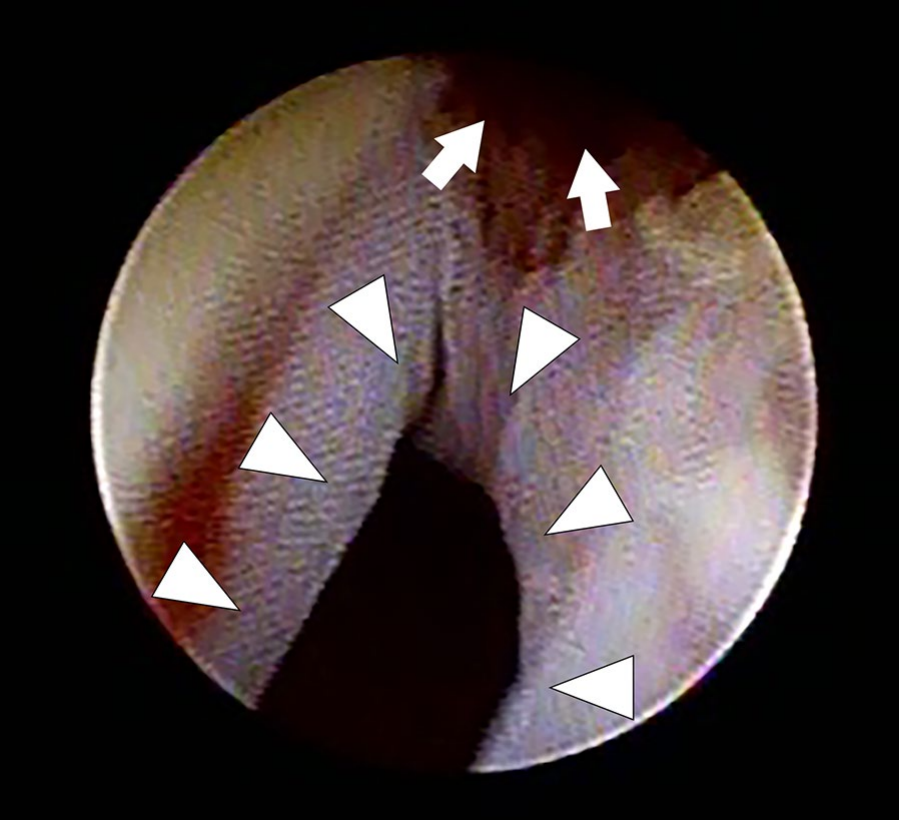
Cystoscope shows a urethrovaginal fistula (arrowhead) and proximal urethra (arrow)

This revealed the presence of a UV fistula approximately 1 cm from the urethral orifice (Fig. 4). The proximal urethra was unobstructed and connected to the bladder. A cystoscope allowed for the passage of a urinary catheter through the urethra. Since suture closure of the UV fistula was expected to be difficult during the neonatal period, an IUC was placed for 1 month to ensure that the bladder would empty and that the upper urinary tract would be safe, and in anticipation of a spontaneous closure of the UV fistula. After removal of the catheter, steady urination was observed, and she was discharged from the hospital at 57 days of age. Follow-up was uneventful, with neither urinary infection nor retention. To check for tumor recurrence, she will undergo a cystoscopic evaluation of the UV fistula and follow-up tumor marker and imaging studies.

## Discussion

SCTs commonly manifest as sizable lesions at birth and can result in various obstructive symptoms as they develop in the presacral region. SCTs comprise 70% of all teratomas in childhood, with a greater incidence in females [4]. Antenatally, approximately 20% of cases exhibit polyhydramnios [5], and approximately 80% can be identified through prenatal ultrasonography scans [6].

SCTs are associated with not only trouble with rectal function and lower extremity movement but also urinary complications. Urologic complications have been reported in 22–46% of all cases [3, 7, 8]. The reported complications are urinary retention, hydronephrosis, neurogenic bladder, vesicoureteral reflux, bladder distension, kidney anomalies, hydrocele, and undescended testes [3]. The causes of these complications are still incompletely understood, and it is controversial whether iatrogenic damage from tumor resection or a mass effect from compression of the developing pelvic structures plays the predominant role in the functional outcomes [7, 9–11].

The association between urogenital (UG) anomalies and SCTs is unclear. Nieuwenhuijs reported a case in which a UV fistula was observed alongside an absent mid-urethra and a circular stenosis of the vagina [3]. Halleran reported a case involving SCT with a UV fistula, including a patent proximal urethra and atrophy of the distal urethra beyond the UV fistula [12]. Some cases of UG anomalies have been reported, whereas cases showing the coexistence of SCTs with remnants of the urogenital sinus have been scattered [13, 14].

Shalaby reported 5 female cases with urogenital anomalies associated with SCT in a national survey in Scotland, none of which had been detected during the initial surgery [15]. UG anomalies were categorized into three types based on the connection between the urethra and the vagina. (1) Both the patent proximal urethra and distal urethra are connected separately to the vaginal cavity, resembling a pi-shaped (π) configuration. (2) The patent proximal urethra is linked to the anterior vaginal wall, with the distal urethra collapsed en route. (3) A patent proximal urethral connection to the vaginal vault, but no identifiable distal urethra, resembling a persistent urogenital sinus. According to this classification, the case reported by Nieuwenhuijs is type 1 and that reported by Halleran is type 2. Our case showed a lateral UV window, wherein both the patent proximal and distal urethra were connected to the vaginal cavity at the same position, resembling a K-shaped configuration. This presentation appeared to fall under type 1.

The pathogenesis of this UV fistula is thought to be the rapid growth of the SCT that developed in the fetal period, resulting in obstruction of the urethra by the tumor and the pubic bone, which in turn caused urinary retention and the formation of a fistula as an escape route for the pressure. The subsequent formation of a hydrocolpos, increased vaginal pressure, and urination through the vulva somehow facilitated urination during the fetal period. The formation of a fistula could be attributed to damage resulting from catheter insertion or other manipulations. However, the patient had a hydrocolpos at birth, and a catheter could not be inserted into the bladder before surgery. Thus, it is likely that a urethrovaginal fistula was present preoperatively. In addition, since catheter insertion was the only preoperative procedure performed on the urethra and there was no hematuria during or after insertion, it is unlikely that the manipulation of the urethral catheter caused the fistula.

The differences between the above-mentioned UG anomalies associated with SCT category seemed to be influenced by the degree and timing of compression. The proximal urethra can be kept patent due to the pressure of urine from the bladder, while the distal urethra is more prone to luminal occlusion and/or regression. Urogenital sinus cases linked with SCT might fall within a group of disorders with a shared etiology, and instances presenting urogenital sinus are classified as type 3.

Due to the heterogeneity of these factors among cases of SCT, a wide range of complication patterns is anticipated. Humbraeus et al. reported that cases of SCTs with complications had significantly higher tumor growth rates and significantly larger tumor sizes at gestational week 20 [1].

Treatment of UV fistula includes insertion of a urethral catheter or surgical closure of the fistula. In our patient, however, surgery would have been difficult during the neonatal period, so she was placed on an indwelling urethral catheter for a relatively long period of time. Spontaneous urination was intermittently observed after catheter removal, with neither infection nor other troubles.

It will be necessary to reevaluate and treat the UV fistula in the future. If the fistula has not closed, a fistula closure procedure or total urogenital mobilization will be performed once it has reached an appropriate size.

In summary, we experienced a female SCT with hydrocolpos and UV fistula. The rapidly growing tumor seemed to compress the urethra and vagina to the pubic symphysis, causing urinary obstruction and retention. The spontaneously formed UV fistula and hydrocolpos formation must have facilitated urination in the fetal period. Tumor excision and a 1-month urethral catheter insertion to ensure bladder emptying with the use of a cystoscope allowed the patient to avoid cystostomy and progress without urinary complications. SCTs can cause a variety of complications depending on the course of the disease. Therefore, careful examination and follow-up are necessary.

## Data Availability

Not applicable.

## Abbreviations

STC: Sacrococcygeal teratoma
UV: Urethrovaginal fistula
IUC: Indwelling urinary catheter
UG: Urogenital
